# Infoveillance of the Croatian Online Media During the COVID-19 Pandemic: One-Year Longitudinal Study Using Natural Language Processing

**DOI:** 10.2196/31540

**Published:** 2021-12-24

**Authors:** Slobodan Beliga, Sanda Martinčić-Ipšić, Mihaela Matešić, Irena Petrijevčanin Vuksanović, Ana Meštrović

**Affiliations:** 1 Department of Informatics University of Rijeka Rijeka Croatia; 2 Center for Artificial lntelligence and Cybersecurity University of Rijeka Rijeka Croatia; 3 Faculty of Humanities and Social Sciences University of Rijeka Rijeka Croatia

**Keywords:** COVID-19, pandemic, online media, news coverage, infoveillance, infodemic, infodemiology, natural language processing, name entity recognition, longitudinal study

## Abstract

**Background:**

Online media play an important role in public health emergencies and serve as essential communication platforms. Infoveillance of online media during the COVID-19 pandemic is an important step toward gaining a better understanding of crisis communication.

**Objective:**

The goal of this study was to perform a longitudinal analysis of the COVID-19–related content on online media based on natural language processing.

**Methods:**

We collected a data set of news articles published by Croatian online media during the first 13 months of the pandemic. First, we tested the correlations between the number of articles and the number of new daily COVID-19 cases. Second, we analyzed the content by extracting the most frequent terms and applied the Jaccard similarity coefficient. Third, we compared the occurrence of the pandemic-related terms during the two waves of the pandemic. Finally, we applied named entity recognition to extract the most frequent entities and tracked the dynamics of changes during the observation period.

**Results:**

The results showed no significant correlation between the number of articles and the number of new daily COVID-19 cases. Furthermore, there were high overlaps in the terminology used in all articles published during the pandemic with a slight shift in the pandemic-related terms between the first and the second waves. Finally, the findings indicate that the most influential entities have lower overlaps for the identified people and higher overlaps for locations and institutions.

**Conclusions:**

Our study shows that online media have a prompt response to the pandemic with a large number of COVID-19–related articles. There was a high overlap in the frequently used terms across the first 13 months, which may indicate the narrow focus of reporting in certain periods. However, the pandemic-related terminology is well-covered.

## Introduction

### Background

Media coverage plays an important role in public health emergencies such as the COVID-19 pandemic and serves as a key communication platform during global health crises [1]. The media represent a bridge between science and society and have great power in forming collective opinions, attitudes, perspectives, and behaviors [2]. Recent studies proposed new disease-spreading models integrating media coverage as a strong factor that may influence human behavior in the context of disease transmission [3-5]. All of these studies confirm that the media may affect the spread and control of infectious diseases. Wang et al [4] explained that media coverage has an impact on the implementation of public intervention and control policies. They pointed out that one of the measures is to educate people and explain how to prevent the disease through all available sources of information.

On the other side, the media, especially internet-based information sources, may cause an infodemic, which is described as an overabundance of information, misinformation, and disinformation. Coping with these phenomena created the discipline of infodemiology [6,7]. Eysenbach [8] defined the four pillars of infodemic management, including information monitoring, or infoveillance, which enables gaining better insight into how the media respond to a crisis.

The infodemic is one of the severe consequences of the COVID-19 pandemic [9,10]. This raises many challenges for the task of infoveillance in terms of massive data sets, such as large communication volumes, new terminology related to COVID-19, various topics and domains present in the media (eg, health care, economy, politics, education), and the large number of users involved in communication in social media. Recently, natural language processing (NLP) technologies have enabled progress in dealing with the large amount of accumulating textual data [11] and thus are promising underlying methods as an integral part of infoveillance methodology.

### Prior Work

The significance and impact of the media in the context of an epidemic has been extensively studied for several epidemics before COVID-19, such as H5N1 influenza [12], severe acute respiratory syndrome (SARS) [13], Middle Eastern Respiratory Syndrome (MERS) [14], H1N1 influenza [15], and Zika virus disease [16]. The outbreak of the COVID-19 pandemic resulted in numerous research publications focused on different aspects of public communication, including the linguistic perspective of the online news media [17], content analysis of global media framing of COVID-19 [18], politicization and polarization in COVID-19 news coverage [19], and amount of media coverage in the context of the pandemic [2]. The studies related to infoveillance have mostly focused on discovering topics [20,21], sentiment analysis [22,23], or fake news detection [24,25].

Most studies employed different NLP techniques for capturing specific aspects of the COVID-19 content published online. For discovering public perceptions, opinions, and attitudes toward specific COVID-19–related topics, researchers commonly combine topic modeling and sentiment analysis [21,26-28], which are also occasionally combined with named entity recognition (NER) [29].

Although COVID-19–related media coverage has been widely studied, there are still some aspects of the task of infoveillance that can be improved. For example, existing studies are largely focused solely on the content of the texts rather than on the volume of published texts. There are only a few exceptions in which the dynamics of publishing have been analyzed [2,20]. Moreover, the majority of analyzed data sets consist of texts published at the beginning of the pandemic, which only capture a short time span of 3 to 4 months. Given the lack of research applying longitudinal data monitoring over larger time spans (ie, the first year of the COVID-19 pandemic), our study might be worthy of attention.

In this study, we followed similar methodologies as described above. However, to more specifically address the mentioned gaps, we propose extensions of these methods contributing to the theoretical framework for the task of infoveillance. First, we combined statistical methods and NLP techniques to track the number of news articles and the content of news articles at the same time. Second, in the proposed approach, we applied the Jaccard similarity coefficient for measuring the similarity of the most frequent terms and entities in COVID-19–related online news articles.

### Goal of This Study

In relation to prior work, we developed an approach for the task of infoveillance based on combining NLP and statistical methods, focused on the content from online news media.

By providing an analysis of the online media’s response to the pandemic, we aimed to contribute to the discipline of information monitoring, particularly to gain a better understanding of: (1) the role that internet-based sources play in communication during the COVID-19 crisis and (2) the potential infodemic. Our goal was to achieve NLP-based longitudinal tracking of the dynamics of changes in the coverage of the Croatian online news space. Noting that the Croatian media are reported as being poorly trusted [30] further motivated us to explore how the media have treated one of the most challenging situations in the everyday life of the country’s citizens.

This study addressed the following research questions related to the period of the first 13 months of the pandemic: (1) What is the number of COVID-19–related news articles and is this number correlated with the number of new COVID-19 cases? (2) What are the main key terms, the most frequent pandemic-related terms, and the most frequent entities in the focus of the online news media? (3) How has the COVID-19–related content (in terms of the most frequent words, most frequent pandemic-related terms, and main entities related to the pandemic) in the online news changed during the first 13 months of the pandemic?

To answer these questions, we performed the following analyses. First, we carried out an exploratory statistical analysis of online media to provide an overview of the trends of COVID-19–related articles published during the first year of the pandemic. Next, we developed a set of statistical and NLP-based methods for the task of infoveillance of the content published on online news media. More specifically, we applied NER for the automatic extraction of the entities that play a key role during the pandemic. Next, we constructed a simple visualization monitor enabling the longitudinal tracking of the change of the pandemic-related terms contrasted between the first and second waves of the pandemic. Finally, we quantified and visualized the changes of the most frequent terms and entities using the Jaccard similarity coefficient over the 13 months.

## Methods

### Data Collection

In this longitudinal study, the collected data covered a period of more than 1 year, specifically the period from January 1, 2020, to January 15, 2021, thereby covering the time period corresponding to the first two pandemic waves in the Republic of Croatia (see Table 1). We included January and part of February 2020 in the study period, although this represents the time before the first reported COVID-19 case in Croatia. With the inclusion of this short period before the pandemic outbreak, the data set contains the emergence of seed pandemic-related terminology. Moreover, the captured antecedent period served as the control for the comparison with the official pandemic period. More details about the duration of the epidemic (pandemic) waves can be found in Section-A1 of Multimedia Appendix 1.

The data were selected among publications from eight mainstream online news media sources, distributed to cover the geographical and media space of the Republic of Croatia. The articles were collected on a daily basis, resulting in 270,359 articles in total, 121,095 of which were COVID-19–related news articles. Collected articles represent the full sample of all articles published in these eight portals in the defined period. We refer to the data set of the COVID-19–related articles as “Cro-CoV-texts2020” (see Multimedia Appendix 1 for a link to the publicly available lists of the word frequencies extracted from all news sources grouped by month). These eight portals included in the Cro-CoV-texts2020 data set do not cover the entire online news media space of Croatia. Nevertheless, they form a representative sample for our longitudinal study. The criteria for their selection are described in detail in Section-A0 of Multimedia Appendix 1.

**Table 1:** Duration of pandemic waves in Croatia.

Period	Start date	End date
First pandemic wave	January 1, 2020, February 25, 2020^a^	May 22, 2020
Pandemic subsides	May 23, 2020	June 14, 2020
Second pandemic wave	June 15, 2020	January 15, 2021

^a^Appearance of the first COVID-19 case in Croatia.

The filter used to determine the affiliation of an article to a COVID-19 class was the occurrence of keywords from the coronavirus thesaurus in the title, subtitle, or body of the text. The coronavirus thesaurus contains approximately 20 of the most important words describing the SARS-CoV-2 virus epidemic, as well as all inflectional variations (see Section-A2 of Multimedia Appendix 1). In addition to the general words (universal keywords related to the COVID-19 pandemic), the list was expanded with additional terms specific to Croatia, including the names of public administration authorities (eg, the Minister of Health, a leading state epidemiologist, the director of the National Civil Protection Headquarters).

The collected articles were preprocessed as follows: (1) only the textual part of the news was retained (related images and videos were discarded), and (2) titles, subtitles, and body of the texts were lemmatized to reduce the inflectional variations of the words as a standard NLP preprocessing procedure.

The epidemiological data related to COVID-19 (ie, the number of newly infected individuals) were obtained from the official government portal. The data are available in Section-A0 of Multimedia Appendix 1 for every day in the period from February 26, 2020 (when the first case of coronavirus infection was confirmed in Croatia), to January 15, 2021.

### Statistical Analysis of Online Media Content Volume

After filtering the collected content according to the defined thesaurus of coronavirus terms, we first determined the ratio of the COVID-19–related and remaining publications. We then performed an exploratory statistical analysis of the COVID-19–related online publications.

Specifically, the time series of COVID-19 daily cases was compared with daily published COVID-19–related articles during the entire period from January 1, 2020, to January 15, 2021. Both time series have the same time resolution and the same length of 110 days in the first wave and 215 days in the second pandemic wave. For time-series data that did not follow a Gaussian distribution, nonparametric tests were used. The standard Spearman correlation coefficient (*ρ*) and Kendall coefficient (*τ*) were used to measure the strength and direction of the association between the two variables: the number of cases and the number of articles.

Additionally, the cross-correlation function (CCF) was applied to quantify a potential association, as well as the time lag between the two time series (see Equation 1 in Section-A3 of Multimedia Appendix 1). The interpretation of the CCF dictates that larger absolute values of cross-correlation at the time lag indicate a stronger association between the two time series. The correlation is considered to be significant when the absolute value is greater than the threshold defined with Equation 3 in Section-A3 of Multimedia Appendix 1.

Another modality of the experiment aggregated the daily data into a 1-week window for both time series, resulting in the resolution of 15 weeks in the first wave and 32 weeks in the second pandemic wave (46 weeks in total), which is also suitable for calculating the CCF.

The autocorrelation function (ACF) was used to calculate the strength of the relationship between a time-series observation and observations at prior time steps, referred to as “lags.” Because the correlation of the time-series observations is calculated with values of the same series at previous times, this is known as a serial correlation analysis. A plot of the autocorrelation of a time series by a lag is often called the ACF, correlogram, or an autocorrelation plot.

The graphs for the ACF of the autoregressive integrated moving-average residuals include lines that represent the significance limits, which are calculated by Equation 4 in Section-A3 of Multimedia Appendix 1. The values that extend beyond the significance limits are considered to be statistically significant at approximately *α*=.05, providing evidence that the autocorrelation does not equal zero [31].

The mutual information (MI) between the new COVID-19 case counts and the number of published articles related to COVID-19 from February 26, 2020, to January 15, 2021, was quantified to further evaluate the mutual dependence of the two time series. The MI was calculated as the expected value of the pointwise MI of the two time series. The calculations of point-wise MI, MI, and normalized MI are defined by Equations 5, 6, and 7, respectively, in Section-A3 of Multimedia Appendix 1.

As suggested by Safarnejad et al [16], the CCF provides an overview of the association between real-world COVID-19 case counts and the published COVID-19–related articles over a certain time period. In our case (for 325 observations and 28 lags), a CCF above 0.116 would indicate a strong association between the two time series. However, the MI complements the CCF and was used to further quantify this association with an exact numerical value.

### Identification of the Most Frequent Terms and Change Dynamics

In the next step, we analyzed the most frequent terms related to COVID-19 and how the vocabulary trends are changing over time. Specifically, we calculated the frequencies of all of the terms in the lemmatized data set. We performed the same analysis in two different time spans: on a monthly level (13 months in total) and for the two pandemic waves. In the analysis by months, the number of time units (days) depends on the total number of calendar days. In the second case, the duration of the pandemic waves was 281 days in total, with the first wave being shorter at 166 days and the second wave stretching over the remaining 215 days. Roughly speaking, the first and second waves can be considered to have lasted for approximately 6 and 7 months, respectively.

Being aware of the fact that other countries might not relate to the recognition and differentiation of pandemic waves, chunking for Croatia is justified by the collected data. The monthly level analysis is certainly appropriate for further comparison with other countries. In the analysis of coronavirus-related concepts, we compared the trends of how the most frequently used terms were changing during the 13 months and across two different pandemic waves by quantifying the Jaccard similarity that indicates the overlap of the terms between two different periods. There are many approaches available for the extraction of key terms [32]; however, we decided to apply a simple approach based on the word frequencies.

### NER Extraction

NER is an NLP task aimed at the extraction of named entities such as people, locations, organizations, and numeric expressions (ie, time, money, dates). NER extraction can be modeled as a text sequence annotation problem. In this case, the conditional random field (CRF) as a nondirected graphical model was trained to maximize the log likelihood, calculated from the conditional probabilities of the output labels’ sequences over the features of the input sequences and CRF states. Performance of NER for Croatia has been reported previously [33] based on an experiment with three named entity classes (organization, person, location) and yielded an F1 score of 89.8%. In this work, we used the NER system trained for the related Slavic languages Slovenian, Croatian, and Serbian [33,34] to automatically extract entities from the large COVID-19–related data set. The implemented NER was a slight modification of the CRF-based reldi-tagger with Brown clusters information added, capable of the recognition of person, person derivatives (adjectives derived from a person’s name), location, organization, and miscellaneous entities.

## Results

### Descriptive Analysis of the Online Newspaper Space

In our previous work, we analyzed isolated online and social media content published in the Croatian language in a shorter time period [35-38]. In this study, we focused on the eight major representatives of online news media by scrutinizing their publications over a significantly longer period (from January 1, 2020, to January 15, 2021). The percentage of COVID-19–related articles was quantified according to the coronavirus vocabulary.

**Figure 1:**
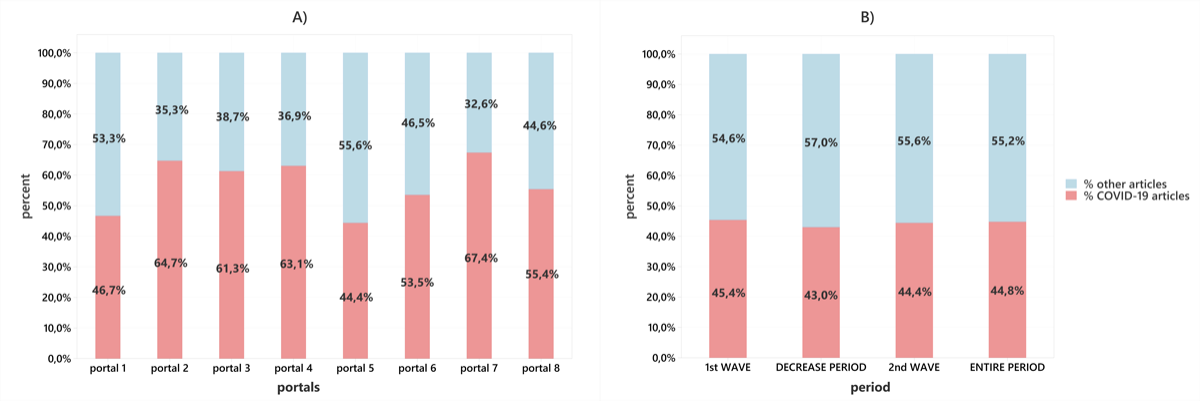
Percentage of COVID-19–related articles summarized for each of the eight online news media sources during the pandemic in Croatia (February 25, 2020, to January 15, 2021) (A), and the percentage of COVID-19–related articles relative to the total number of articles summarized across the eight online news media sources for different periods during the pandemic (B).

The percentage of COVID-19–related articles did not fall below 44% for any of the eight observed online news media sources (Figure 1A). The average ratio across all online news media sources of COVID-19–related publications occupied more than half of the total media space (approximately 57%).

Figure 1B shows the percentage of COVID-19–related articles relative to the total number of published articles, summarized for all eight online news media sources for different time periods (ie, the two pandemic waves, the period in which the pandemic subsided marked as the decrease period, and for the entire period of 13 months). To gain a global picture for 2020, data from January 1 to February 25, 2020, were also analyzed despite the fact that there were no cases of COVID-19 in Croatia in that time. The percentage of COVID-19–related articles in the first wave would take on a much higher value if the analysis did not include days in which there were no cases of infection in Croatia (the period from the beginning of the year to February 26, 2020) and would rise to a value of 57%. Surprisingly, in the period between the two pandemic waves, when the number of cases of infection dropped to zero (the decrease period), the number of publications related to COVID-19 remained high at 43%, despite expectations that the media would write significantly less about COVID-19.

### Association Between the COVID-19 Pandemic and Writing Dynamics in the News Media

Many factors may influence the increased interest in COVID-19–related issues in the media, including the number of patients on mechanical ventilation due to deterioration of their condition, the number of people in self-isolation, the daily or total number of deaths from COVID-19, and the number of companies and entrepreneurs who had to stop their regular business due to the pandemic. The testing of all of these claims was impeded by the unavailability of reliable data. Nevertheless, we examined an isolated variable with potential to influence COVID-19–related publications and from which we could obtain reliable data. Hence, we aimed to determine whether there is a correlation between the number of daily cases of newly infected people with SARS-CoV-2 and the number of published news articles related to the topic of COVID-19.

**Figure 2:**
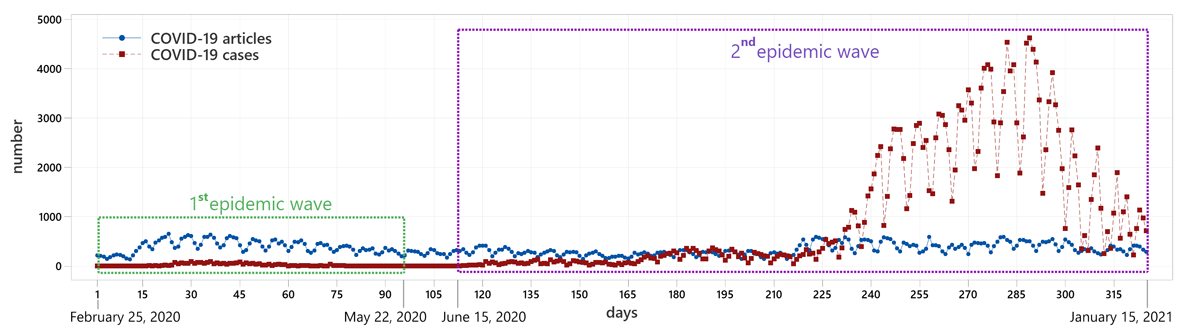
Time-series plot comparing the number of published COVID-19–related articles per day (blue) and the number of new COVID-19 cases (red) from February 25, 2020, to January 15, 2021.

The time-series plot in Figure 2 shows the number of new COVID-19 cases per day (red line) and the number of published COVID-19–related articles (blue line). The blue line has the same pattern of wavy repetition throughout the observation period, regardless of the epidemic wave, whereas the red line has an elongated left tail and then a high ridge in the second epidemic wave. In addition, slight repetitive wave-like oscillations can be seen along the time axis (days). Data distributions are shown with the histograms of the frequencies for both observed time series in Figure A4-1 of Section-A4 in Multimedia Appendix 1.

We next examined whether there is a linear relationship between the number of new cases of COVID-19 per day and the number of publications of COVID-19–related news articles per day using the Spearman rank correlation coefficient. The null hypothesis was that there is no correlation between the number of COVID-19 cases and the number of published articles related to COVID-19 (*α*=.05), which was rejected given a weak but statistically significant correlation (n=325; *ρ*=0.253, *P*<.001); this was additionally confirmed with Kendall *τ*=0.173 (*P*<.001). More detailed results, including the 95% CIs for the 2-tailed test, are reported in Section-A4 of Multimedia Appendix 1.

Although statistically significant, the correlation was extremely weak. To obtain a direct interpretation of results, we used the Kendall *τ* coefficient in terms of the probabilities for observing the agreeable (concordant) and nonagreeable (discordant) pairs. The ratio of the occurrence of concordant to discordant pairs was 1:1.4 (ie, 1+*τ*/1–*τ*), which means that the probability of occurrence of concordant pairs is 1.4 times higher than the occurrence of discordant pairs.

Realistically, it is to be expected that the number of publications on the topic of COVID-19 will not increase on the same day as the number of COVID-19 cases increases (or decreases), but that the media will write about it subsequently (ie, the next day or a few days later). Therefore, we next examined whether the correlation can be stronger if we observe the publication of COVID-19–related articles with a time delay compared to the daily number of COVID-19 cases.

Given that cycles can be seen in the time-series data that repeat regularly over time in the form of a sine wave (see Figure 2), this could represent seasonal variations. However, a cycle structure in a time series may or may not be seasonal. Correlograms in Section-A4 in Multimedia Appendix 1 show plots of the ACF on time-series data of new COVID-19 cases (left plot) and published COVID-19–related articles by a lag (right plot). This autocorrelation measures the linear relationship between the lagged values of a time series. The ACF for both COVID-19–related articles and new COVID-19 cases showed several significant peaks after a lag of 7 days. This determines the cyclic behavior in the time-series data in which the cycles are repeated every 7 days. The reason for this is that on nonworking days (ie, Saturday and Sunday), less news is written and published and thus a minimum cycle value is achieved. By contrast, during working days (usually in the middle of the week), there is a larger number of published news articles (ie, the maximum cycle value is reached). It is important to emphasize that there is no complete regularity in the cycles (ie, there is no seasonality on a 7-day basis). The reason for this is that the maximum number of news articles does not always occur on the same day of the week. The peak can shift among Tuesdays, Wednesdays, or Thursdays. The same holds for the number of new confirmed cases of COVID-19. On weekends, a smaller number of people are tested (corresponding to the same days when less news is published), whereas more people are tested on work days, so that the number of confirmed infections is higher. The peak is reached again in the middle of the week, but not always on the same day, so the regularity in the form of seasonality cannot be credibly confirmed for the entire epidemic year. The results could suggest the presence of a weekly seasonal component for certain shorter periods of the year. Finally, for the entire year, we observed with certainty a cyclical behavior on a weekly or 7-day basis.

According to these insights, we aggregated the data on the time series by week (7 days), and observed them in the 1-week time window. The Kolmogorov-Smirnov normality test showed that the data do not follow the Gaussian distribution (test details are available in Section-A4 of Multimedia Appendix 1). Again, the null hypothesis was that the correlation does not exist (*α*=.05). Spearman correlation of ranks indicated the existence of a slight positive correlation (n=47; *ρ*=0.277), which was slightly higher than that obtained in the previous case analyzed on a per-day basis, but was not statistically significant (*P*=.06). Additionally, this was confirmed with Kendall *τ* of 0.202 (*P*=.05); the 95% CIs are reported in Section-A4 of Multimedia Appendix 1.

**Figure 3:**
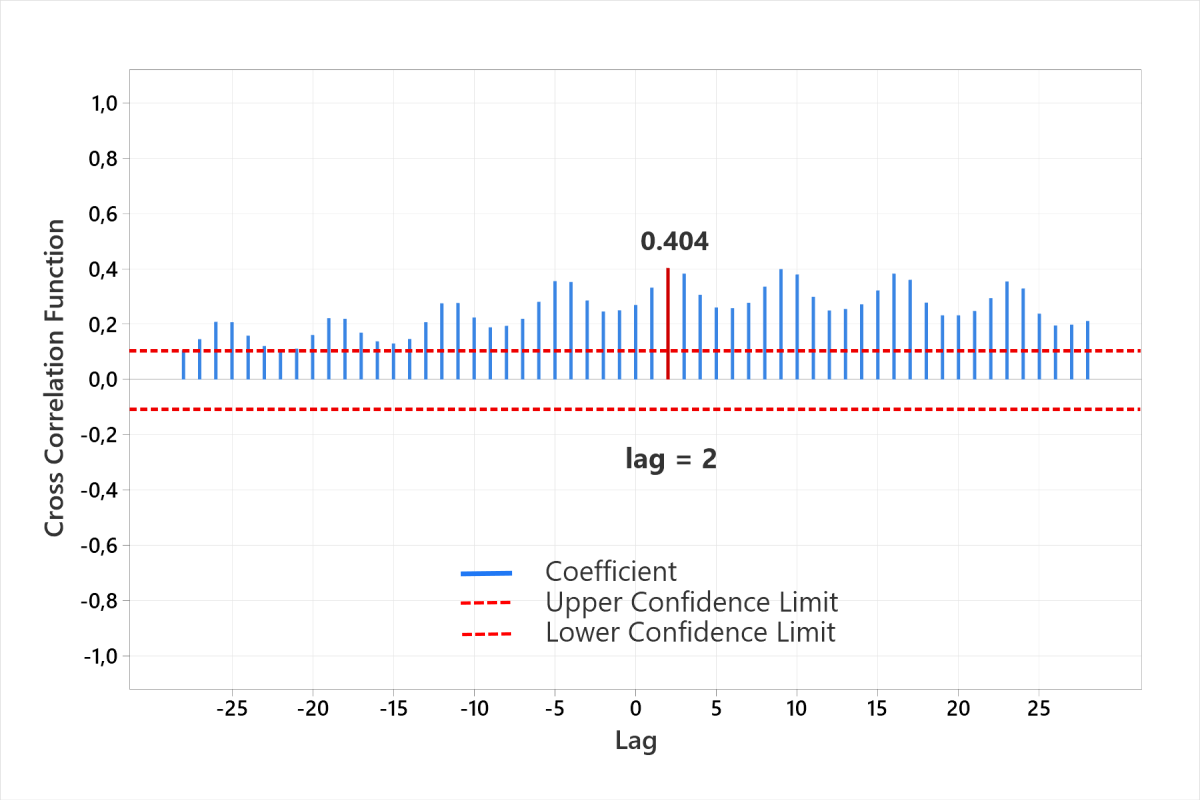
Cross-correlation function between the published COVID-19–related article counts per day and the number of confirmed COVID-19 cases per day.

Due to the vague picture of the existence or nonexistence of at least a weak positive correlation, we performed an additional cross-correlation test on the time-series data measured on a daily basis. A significant cross-correlation between the published COVID-19–related article counts and the number of confirmed COVID-19 cases per day was observed for the pandemic in Croatia (Figure 3). The CCF was substantially above the threshold of statistical significance, and the strongest positive correlation occurred at lag=2. This shows that the two variables are not contemporaneously correlated. However, the positive correlation at lag +2 suggests that higher numbers of COVID-19 cases lead to higher numbers of published articles related to COVID-19 themes 2 days later. Negative correlations were not detected in the observed lag range.

Cross-correlation tests indicated that publishing COVID-19–related news articles was not completely decoupled from the actual disease pandemic in the Republic of Croatia’s online news space. This indicates the underlying effect of the COVID-19 pandemic on the writing about COVID-19. Finally, the strong dependence between the two time series was further quantified and confirmed by MI and the normalized MI measure (for details see Section-A4 of Multimedia Appendix 1).

Next, we asked whether there is a linear relation among the eight major online news media sources considering the number of COVID-19–related articles published per day. For all 28 possible cases, the correlations were statistically significant. In terms of the Spearman coefficient, all correlations were positive and a correlation was absent in only two cases. Furthermore, in 12 cases, the positive correlation was weak, in the next 12 cases, it was substantial, and in 2 more cases, it was strong. The correlations were confirmed with Kendall *τ* as a more conservative coefficient (see Section-A4 of Multimedia Appendix 1).

### Pandemic-Related Terminology Analysis

The analysis of the most frequent terms was performed at the granularity of pandemic waves. The top eight highly frequent terms in the first and in the second epidemic waves were found to be identical, according to their frequency in COVID-19–related media releases. This is an indication that throughout the pandemic year, regardless of the epidemic wave, journalists most often mentioned the following terms: people, coronavirus, Croatia, year, measure, day, high/large, and new. This represents an extremely narrow vocabulary with a small set of three terms that consistently refer to the epidemic year in Croatia and five more terms that are used daily in the news describing the high daily number of newly infected people.

**Figure 4:**
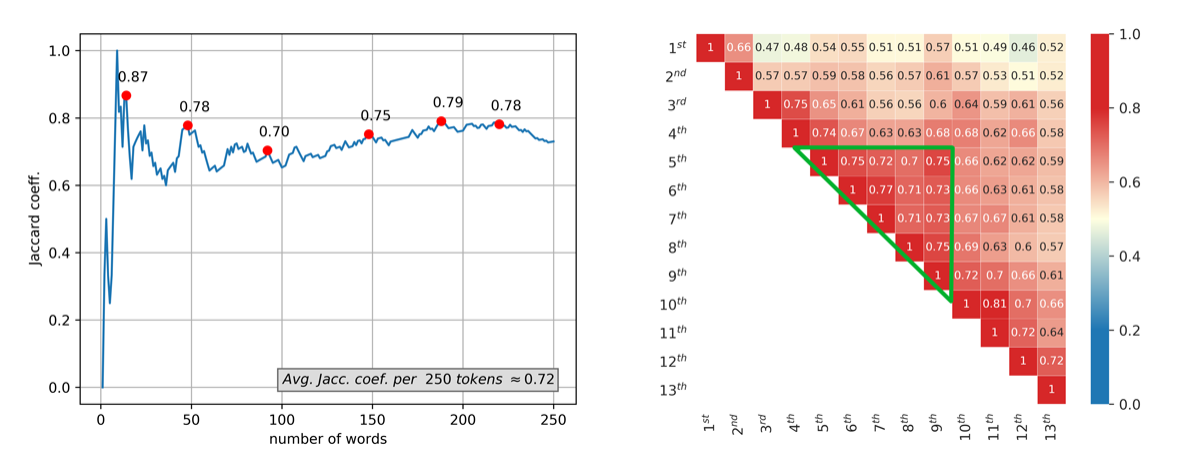
Jaccard similarity coefficients of the most frequent words (terms) between the first and second waves (left) and among the 13 months of the COVID-19 pandemic in Croatia (right).

Expanding the monitored list to the top 250 most frequent terms during the first and second epidemic waves showed an average Jaccard similarity coefficient of 0.72 (see the curve oscillations in Figure 4, left). This is an indication of a significant overlap of the most frequent terminology between the pandemic waves, and hence the consistent content of pandemic-related writing in online media. Table A5-1 in Multimedia Appendix 1 lists the 50 most frequent terms used in news publications for the first and second epidemic waves, respectively.

In the second step, the terminology analysis was performed at the month granularity. The Jaccard similarity coefficient was calculated for the 250 most frequent terms between every two months. The heat map in Figure 4 shows significant deviations in January and February (yellow squares), followed by some high overlaps in terms of the Jaccard similarity (red squares). The green triangle on the heat map indicates the period with the highest overlap (ie, the most used terminology in those months was the most similar). All angles of the green triangle have a value of 0.75 and thus delimit the months in which the epidemic subsided and people lived with less pressure from infection. During the “green triangle” months, the media virtually revolved around the same most frequent pandemic terms, and the reasons are related to two upcoming events: parliamentary elections and the tourist season. Moreover, the prime minister, who was running in the elections, announced that the income from tourism, which is always important for the Croatian gross domestic product, would be crucial during the pandemic.

**Figure 5:**
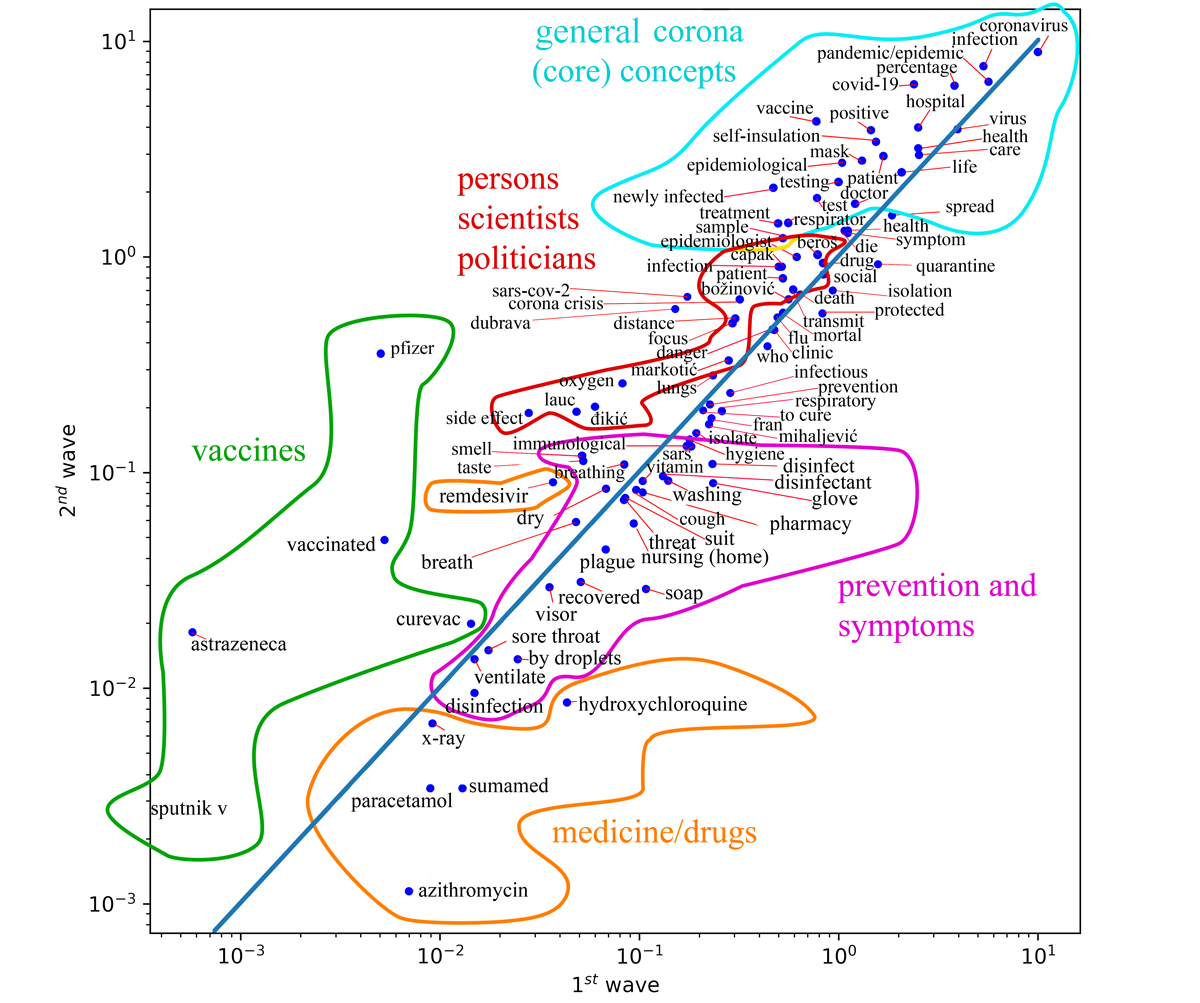
Relationship of epidemiological vocabulary between the first (lower right) and second (upper left) pandemic waves.

The prevalence of pandemic terminology in the first and second epidemic waves was quantified and is visualized in Figure 5. The terms below the blue diagonal line are those that were more frequently identified in the media during the first wave and the terms above the line are those that were more frequent during the second wave.

According to the results, the symptoms that were more common in the first wave were cough, sore throat, and respiratory symptoms, whereas writing about symptoms during the second wave was more focused on the lungs and breathing, taste, smell, and dry cough. It is important to note that the differences in frequencies between all of these terms are small and that they were written about at almost equal rates in both waves. The symptoms of anosmia, ageusia, and parosmia appeared with the highest occurrence frequency.

The necessities for maintaining hygiene and preventing the spread of infection were predominantly mentioned in the first wave, including disinfectants, gloves, soap, visors, and even the pharmacies that trade in such supplies. The next important group of terms was related to drugs. Azithromycin (Sumamed), paracetamol, and hydroxychloroquine were mentioned more in the first wave. In the second wave, once we gained more knowledge about the disease, remdesivir was more frequently mentioned, accompanied by the rise of vaccination-related terminology (eg, CureVac, Pfizer, AstraZeneca, and Sputnik V). In addition, the word “vitamins” was frequently identified, as well as “oxygen” due to intensification of the pandemic outbreak in the second wave.

Subjects from the political scene, such as the minister of the interior affairs (Božinović) or the minister of health (Beroš), the director-general of the Institute of Public Health (Capak), and the director of the largest Clinic for Infectious Diseases in Croatia (Markotić), were more frequently mentioned in the second wave. Scientists (eg, Lauc and Đikić) were more frequently mentioned in the second wave because they made more media appearances at that time. Nevertheless, politicians were mentioned more often than scientists.

During the first wave, more attention was paid to the ways of spreading the disease and infection prevention. Therefore, terms such as spread (infection or disease), isolation, quarantine, infection, and disinfection were mentioned more often in this wave. Interestingly, the terms “self-isolation,” “newly infected,” “infection,” “transmission,” “treatment,” “sample,” “positive test,” “testing,” “epidemiologist,” “social distance,” “to die,” “patient,” and “mechanical ventilation (respirator)” had a significantly higher incidence in the second wave. This might be a result of a significantly higher number of infections in the second epidemic wave, which was magnitudes higher than that in the first.

Among the terms that refer to diseases, “the plague” and “SARS” prevailed in the first wave, whereas “influenza,” “SARS-CoV-2,” and “COVID-19” dominated in the second wave.

General words used for describing COVID-19 infection and disease such as “virus,” “coronavirus,” “infection,” “hospital and health care,” “pandemic,” “epidemic,” “life,” and “patient” are immediately close to the wave-dividing boundary. Due to their generality, their frequency was magnitudes higher than the frequency of terms that describe or name symptoms, medications, public figures, medical institutions, and similar.

We paid particular attention to drugs and vaccines that were most frequently mentioned at the time of the pandemic. The details of the observed word groups naming drugs and vaccines can be found in Table A5-2 in Multimedia Appendix 1. The representation of the groups in the corpus is expressed as a percentage.

Results are reported separately for drug and vaccine terminology as normalized values for the first and second waves. The group of drug-related words occupied 0.38% of the corpus from the first wave and 0.61% of the second wave. The group of vaccine-related words occupied 0.24% and 4.63% of the corpus in the first and second wave, respectively. The occurrence of words from both groups increased in the second epidemic wave: 0.23% more terms referred to drugs in the second wave than in the first wave, and as many as 4.02% more terms referred to vaccines in the second wave. In the first wave, existing drugs that could help treat COVID-19 were reported, but with the emergence of some new drugs (eg, remdesivir), their mention in the second wave was relegated to the background. As the production of vaccines was announced mainly during the second wave, vaccine-related reporting became more exhaustive.

### The Main Subjects in the Pandemic

**Figure 6:**
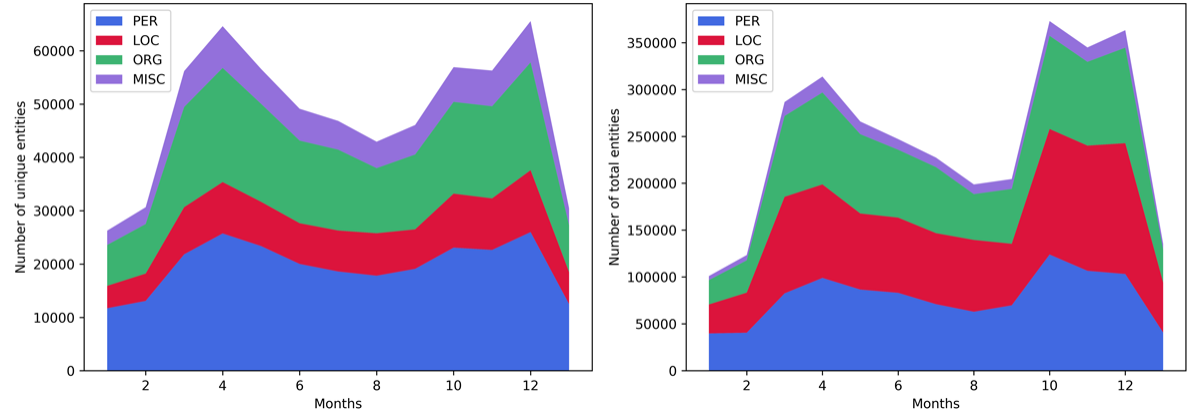
Ratio of the representation of unique entities (left graph) and the total number of recognized entities (right graph) in COVID-19–related media releases in summary for all observed online news media. PER: person; ORG: organization; LOC: location; MISC: miscellaneous (general).

Analysis of the ratio of unique entities and the total number of entities in the pandemic articles was obtained by NER. The results indicated that the proposed longitudinal tracking of focal entities can serve as one aspect of infoveillance, providing insights into the trends of public interest. Figure 6 reveals that the numbers of people and organizations were significantly higher than those of locations and general (miscellaneous) entities. Combined with the insights from Figure 6 (right), where the total number of detected entities in the categories people, organizations, and locations were fairly equal and the miscellaneous category was marginal, it is possible to enable the consistent tracking of the public interest during the pandemic. The left part of Figure 6 provides numerical insight into the representation of individual subjects in media coverage during the pandemic. In the first pandemic year, among the four studied groups of entities, people (the blue graph area) were the most frequent subjects in coronavirus-related news. In addition to personal names, nationalities also belonged to a group of entities collectively referred to as “person.” The personal names mostly referred to leading figures of the political scene, the presidents of the state and government, the heads of the civil protection headquarters, ministers, scientists, hospital directors, and infectious diseases specialists.

The second most frequent group was organizations (green). During the pandemic year, most journalists wrote about hospitals, public health schools, testing centers, civil protection headquarters, the World Health Organization (WHO), European Medicines Agency, vaccine companies, and, surprisingly, the most popular social networks such as Facebook and Twitter; occasionally, these sources referred to football or sports clubs organizations, whereas political organizations and parties were most frequently mentioned.

Locations was the third group of entities (red), including states, cities, and counties. The captured location entities involved the foci of the epidemic or areas where important pandemic-related events were happening, including where the first vaccines were available, antimasker protests, areas running out of oxygen for clinical treatment, infection entering nursing homes, state borders closing, borders opening for the tourist season, schools closing, presidential elections, and a massive earthquake that occurred twice in 2020 coinciding with the pandemic waves (during the first wave it occurred in Zagreb, the capital of Croatia, and during the second wave it occurred in the towns of Sisak and Petrinja in the vicinity of Zagreb). During the pandemic, the news articles mentioned only a limited and consistent set of locations since not much traveling and migration were allowed. Hence, the number of locations was constantly below the numbers of people and organizations.

The last rank was occupied by the group of general or miscellaneous entities (violet). This category includes the names of events, commercial products and brands, documents, TV channels, viruses, and diseases, among others. Their occurrence was highly dependent on the time of year or month in which an event, competition, concert, or promotion takes place.

Finally, both graphs in Figure 6 show that maximal values were reached during the peaks of the first and the second waves of the pandemic.

**Figure 7:**
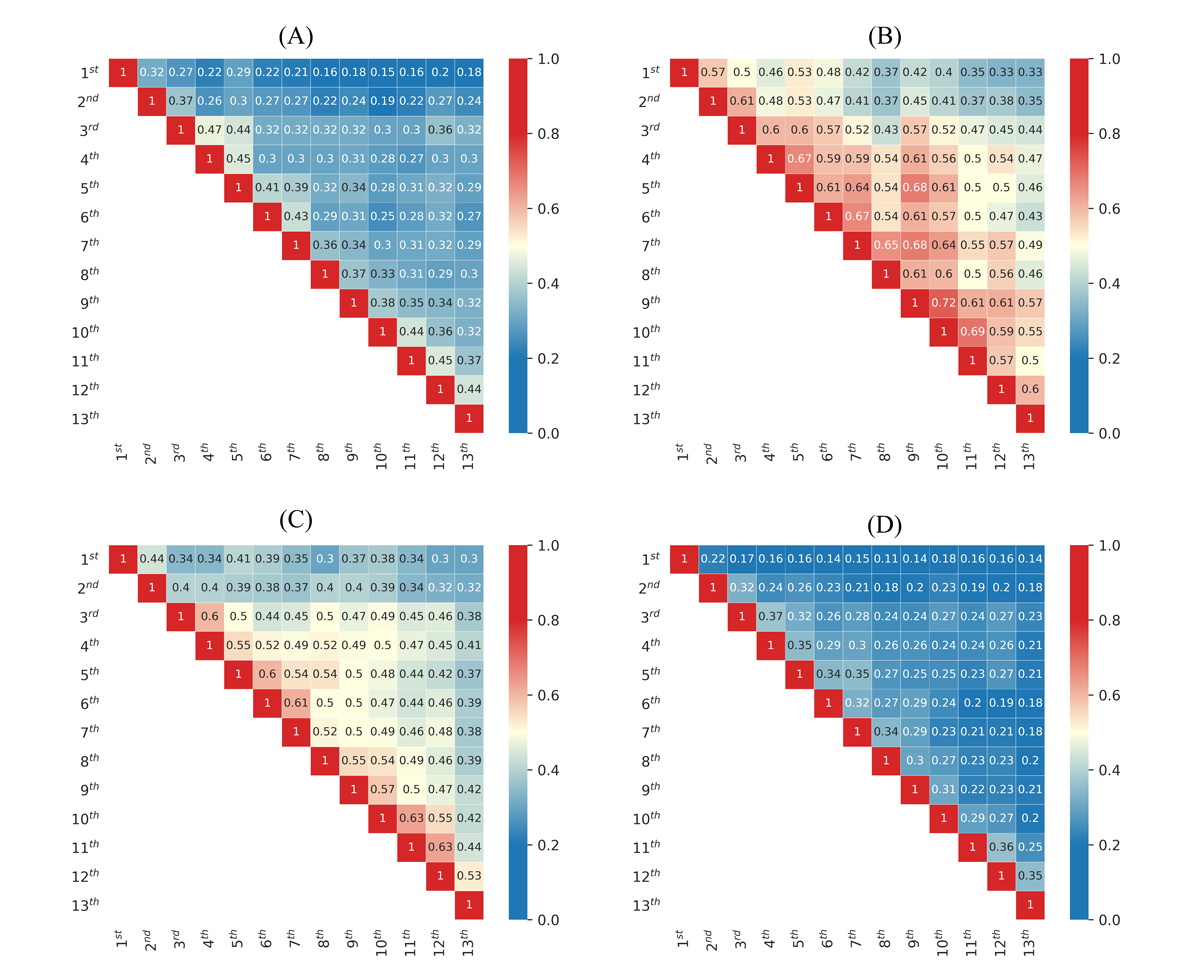
Jaccard similarity coefficients between the 13 months for the 100 most frequent entities per four traditional categories: person (A), location (B), organization (C), and other general entities (miscellaneous) (D).

Quantifying the similarity of the top 100 entities by months during the observed pandemic period, the heat map in Figure 7 reveals higher similarity (Jaccard) values between sets of people and locations than between sets of organizations and miscellaneous entities. The bright red color indicates a stronger overlap, whereas the dark blue indicates disjunction (ie, no overlap) between the observed entities. The yellow color indicates only a mediocre overlap.

Locations showed the highest overlap, whereas person and miscellaneous entities showed the lowest overlap across the months. This indicates that news was dispersed across many people appearing in daily events. In contrast, locations were fairly constant during the pandemic due to the low number of total locations. These results indicate that the focus was on a narrow area restricted to Croatia, the neighboring countries, the European Union, and international locations such as Wuhan and Lombardy. This reflects the fact that countries closed their borders and the majority of events took place inside the country. That is why Croatian cities and regions were the predominant location entities throughout the study period. Similar observations were made for organizations as well. The principal organizations of focus were the WHO, local infectious disease clinics, and hospitals. Besides medical institutions, the focus was on government entities such as the national headquarters, ministries, the Croatian parliament, and political parties. In the second wave, the focused entities were related to vaccination. The names of the most popular social networks (ie, Facebook and Twitter) were also always present because news articles were reporting COVID-19–related discussions on Facebook and Twitter. A difference can be noticed (Figure 7) for January and February 2020 (when the epidemic had not yet been declared in Croatia). These fields are in shades of blue, which indicates that online news media wrote about different organizations until the epidemic broke out. After that (from March 2020 onward), the color changes to yellow and slightly red. The online news media then predominantly wrote about the same set of organizations for the entire duration of the pandemic, even in the month of June in which there was a break between the two epidemic waves.

Furthermore, we performed an entity analysis between the two pandemic waves. In this case, we focused on the 250 most frequent entities per entity type (person, location, organization, and miscellaneous) and observed their overlap between the two epidemic waves. The Jaccard similarity coefficients showed the largest overlaps for the location entity type (0.5337), which was slightly lower for organizations (0.4793) than for people (0.4045) and was the lowest for the miscellaneous (0.333) category. The interpretation of the results is identical to that described above for the analysis by months.

## Discussion

### Principal Results

In this work, we characterized the online media response to the COVID-19 pandemic in Croatia by examining the amount and the content of news articles related to COVID-19. Since most of the studies dealing with the media response to previous world epidemics were performed without using NLP for the task of infoveillance (eg, [12-16]), our study is not fully comparable with this previous work. In response to the other infoveillance studies related to COVID-19 media coverage [20-23,26,27,29], this study offers methodological extension. Specifically, we propose an integrative infoveillance approach based on NLP methods combined with the Jaccard similarity coefficient for longitudinal tracking of the dynamics of changes across the first 13 months of the pandemic.

Our results show that the number of COVID-19–related articles was relatively high, representing approximately 40% of total news articles, on average. This property remained the same during both waves of the pandemic. These results differ from those described by Pearman et al [2], who showed that COVID-19 media coverage decreased after the initial intense attention at the beginning of the crisis. It seems that the online news media in Croatia tended to highly focus on the pandemic during both waves, as well as during the period after the first wave (which, in the following 3 weeks, turned out to be a break before the second wave).

The high amount of pandemic-related articles is one of the three indicators of dramatized media coverage [15], which may indicate an infodemic. However, this alone is not a sufficient condition to confirm an infodemic. Clearly, during the first wave, it was necessary to inform the public about the COVID-19 pandemic. The online media play an important role in informing the public, and perhaps this is the main reason for the high number of COVID-19–related articles despite the relatively lower number of COVID-19 cases during the first wave. Consequently, our findings show that there is no strong correlation between the number of news articles related to COVID-19 and the number of new cases of COVID-19. This finding is in line with a previous study [26] showing that Zika-related tweeting dynamics were not significantly correlated with the underlying Zika epidemic. Additionally, we found that the number of articles and the number of new COVID-19 cases repeated in cycles within the time window of 1 week. There was a constant pattern: the number of articles was smaller during the weekends, and fewer new cases of COVID-19 were reported on Sundays and Mondays.

Capturing the dynamics of changes in the most frequent terms across the 13 months showed the highest similarities from May to September 2020. This was the period with a lower number of COVID-19 cases and it is probable that the news articles were less informative and featured similar topics. Additional examination of the similarities between pandemic-related terms indicated that all of the general terms (such as coronavirus, infection, pandemic, and hospital) were equally present in both waves. The pandemic-related terminology shifted from some possible remedies and medicines that could be used to prevent or cure COVID-19 (eg, disinfectant, paracetamol, Sumamed, azithromycin, hydroxychloroquine) in the first wave to the vaccination process (Pfizer, AstraZeneca, Sputnik V, vaccination) in the second wave. This can be interpreted as a sign of adequate online media coverage in the sense that the online media provided the available information.

The results of NER showed that the online news media concentrates mostly on the people from the state administration; even the scientists featured are often involved as members of the various state bodies. A similar pattern was reported by Hart et al [19], showing that politicians appear in media coverage more frequently than scientists. The online news media showed low dynamics of changes regarding the locations, whereas people, organizations, and other entities were frequently changing over the monitored months.

The inclusion of NER as a method for infoveillance enriches the longitudinal tracking of the dynamics of changes by introducing the insights of focal entities. However, this approach is not a replacement for the topic modeling that is also used as a part of infoveillance methodology [39,40]. In fact, owing to its certain advantages, NER can be a complementary approach to the characterization of the content of information sources. In contrast to topic modeling, which relies on the annotator’s viewpoint and thus raises potential ambiguities in detecting and naming the topics along with challenges regarding interannotator agreement or consistency [32], NER enables unambiguous monitoring since there is no need for an additional interpretation of annotation, which clearly speaks in favor of NER as the complement method of topic modeling.

To the best of our knowledge, this longitudinal study is the first of its kind to use NLP techniques in combination with Jaccard similarity for tracking the changes in the most frequent subjects. In addition, since this study was oriented to the Croatian online news media response during the first year of the pandemic, it can provide useful data for further comparisons with data collected from other countries.

### Limitations

This research has several limitations. First, we characterized media content related to the COVID-19 pandemic by considering only Croatian online news media. However, a large amount of information is present in social media, especially the social networks that were not included in this study. Additionally, individuals are also exposed to COVID-19–related information through traditional sources. Therefore, to obtain a more realistic picture of media content related to the pandemic, it would be advisable to extend the analysis to cover all sources. Hence, in future work, we plan to extend this study by integrating heterogeneous data sources such as online social networks and similar social media platforms, online forums, and all other sources of textual data in social media such as user comments on online news media. Second, this study focused only on the Croatian language; however, the same longitudinal approach can be applied to any other language and/or country, and the entire methodology is transferable and only dependent on the available data sources and the maturity of the NLP methods per selected language.

Furthermore, there are many possible extensions of the reported research. For example, in the inferential statistical analysis, we used only one variable (the number of new COVID-19 cases), but there are also some other variables (eg, number of deaths, number of hospitalizations, number of patients in the intensive care unit or on a respirator) that can be studied as potentially related to the number of published articles. Moreover, several NLP methods can be applied to infoveillance (eg, topic modeling combined with polarity of the sentiment or attitudes in comments). Another important direction of our future research is to develop a full stack of NLP-based methods focused on longitudinal monitoring of the infodemia, infoveillance, health-crisis communication, and infodemic management.

### Conclusion

The presented approach enables the infoveillance of online media in response to the COVID-19 pandemic through quantification of the share of COVID-19–related articles. Specifically, in this study, we addressed three open research questions and our main findings are as follows.

The low correlation between the number of COVID-19–related articles and new cases indicates that the amount of media content is not driven solely by the number of new COVID-19 cases, but rather by external processes. In the first wave, the large amount of news articles was necessary to inform the public about the new disease and the pandemic outbreak. In the second wave, the large number of news articles was important to communicate findings such as vaccines and other epidemiological measures.

Deeper insights can be obtained by analyzing the media content. Quantification of the dynamics of the changes captured by the Jaccard similarity coefficients revealed that there are slow changes in key terminology, locations, and institutions. The similarity between the most frequent terms was higher than 50% across all of the observed months (except for January 2020) and was higher than 70% from May to September 2020. This may indicate the narrow focus of reporting by online media during certain periods. However, additional analysis of the frequencies of the pandemic-related terms between the two waves indicated that there was a shift from the initial medical terminology known in the first wave to the novel medicine approaches and vaccines in the second wave.

To conclude, the online media had a prompt response to the pandemic in the sense of quantity (the number of articles) in both waves that occurred during the first 13 months of the pandemic. Despite the high number of COVID-19–related articles, the key terms and entities encountered slow changes. However, the results based on tracking the dynamics of the changes of pandemic-related terminology suggest that the media covered the important changes during the pandemic (eg, the number of infected people, prevention measures, vaccine production).

Overall, the proposed infoveillance approach based on NLP for longitudinal tracking of the dynamics of changes enables gaining deeper insight into the online news media response to the pandemic. This study thus contributes a better understanding of the published content related to COVID-19 in the Croatian online news media and can be further exploited for improving crisis communication.

## Appendix

Multimedia Appendix 1 Additional information on data, list of COVID-19–related terms used for article filtering, epidemiological data and used data sets, mathematical formulas used in experiments, result details (additional tables and graphs) of inferential statistical analysis, and obtained table of coronavirus-related concepts.

## Abbreviations

ACF: autocorrelation function
CCF: cross-correlation function
CRF: conditional random field
MERS: Middle East respiratory syndrome
MI: mutual information
NER: name entity recognition
NLP: natural language processing
SARS: severe acute respiratory syndrome
WHO: World Health Organization
