# Supplementary material for: Infoveillance of the Croatian Online Media During the COVID-19 Pandemic: One-Year Longitudinal Study Using Natural Language Processing
Source: JMIR Public Health Surveill. 2021 Dec 24;7(12):e31540. doi: 10.2196/31540 (PMC8715984; doi:10.2196/31540)
Supplement: Multimedia Appendix 1 [file publichealth_v7i12e31540_app1.docx]

**Section-A0: Data**

Epidemiological data used in analysis which relates to the number of COVID-19 cases in Croatia for the period from February 26, 2020 to January 15, 2021 was downloaded from the official Republic of Croatia government website for accurate and verified information on coronavirus - *koronavirus.hr*. The data are additionally adapted to comma separated values (CSV) format suitable for experimental analysis. Due to reproducibility and transparent analysis, it is publicly available from <https://github.com/sbeliga/InfoCoV/tree/main/JMIR/StatAnalysis> and organized in three separated datasets:

- **CRO_COVID-19_CASES_DAY.csv** contains data about COVID-19 cases in Croatia isolated by days together with data on the number of published COVD-19 related articles , while
- **CRO_COVID-19_CASES_WIN.csv** contains data aggregated in a 7-day time window together with data on the number of published COVD-19 related articles, besides
- **CRO_COVID-19_PORTALS.csv** contains data of published COVID-19 related articles per day for each portal separately.

Corpora of all newspaper articles used in the analysis, are lemmatized and organized in the form of bigrams with corresponding frequencies, entitled as **Cro-CoV-texts2020.** Dataset is publicly available from <https://github.com/sbeliga/InfoCoV/tree/main/JMIR/Cro-CoV-texts2020>. The data are sorted into separate files and categorized by months. Dataset does not contain unigrams that are treated as stop words in the Croatian language.

All experiments concerning natural language processing and text mining were performed in the programming language Python 3.8. Exploratory analysis and inferential statistical analysis were performed in Python 3.8, Minitab 20.2 and SPSS 26.0.

The selection of eight researched sources (contained in **CRO_COVID-19_PORTALS.csv**) was based on the criteria of:

1. well-known, most popular, viral online news media in Croatia,
2. covering the Republic of Croatia as a whole (i.e. sources that are narrowly oriented to regional news are excluded),
3. reporting on standard categories of news: basic daily news, business, economy, politics, crime, sports, entertainment, art, show business, health, religion, tech, etc.

Taking into account the set criteria, five online news portals (Vecernji.hr, Tportal.hr, Net.hr, Telegram.hr, 24sata.hr) were easily identified from Gemius' list (gemiusAudience, <https://rating.gemius.com/hr/tree/8>) of the most popular Internet portals, and omitting those among them that are not news portals.

Although we noticed in everyday life a respectable public perception of two more media (Index.hr and Jutarnji.hr), we still needed some data that can support the immediate experience. Unfortunately, since 2014 Gemius does not have permission to research the ranking of Index.hr, so we had to rely on previous data, which confirmed that it was indeed one of the most popular online news media. For Jutranji.hr, which is also missing from Gemius’ list, we turned to the ranking of their printed form, assuming that its virality will not be much different in an online form.

According to the survey by the Croatian Competition Agency (Agencija za zaštitu tržišnog natjecanja, <http://aztn.hr>), in 2019 and 2020 its printed form was in third place on the list of best-selling printed newspapers. The first and second place belongs to the print edition of 24sata and Vecernji, both on our list of the most popular online news media. Agency reports can be downloaded from the following links: the report from June 2021 – for the year 2020 – in short form: <http://www.aztn.hr/na-trzistu-tiska-i-dalje-prisutan-visegodisnji-jace-izrazen-trend-pada-prodanih-naklada/>, as well as in full form: <http://www.aztn.hr/ea/wp-content/uploads/2016/10/Istra%C5%BEivanje-tr%C5%BEi%C5%A1ta-tiska-za-2020..pdf>, and the report from June 2019 – for the year 2019 – in short form: <http://www.aztn.hr/na-trzistu-tiska-prisutna-stagnacija-i-nastavak-pada-prodanih-naklada/>, and in full form: <http://www.aztn.hr/ea/wp-content/uploads/2016/10/Istra%C5%BEivanje-tr%C5%BEi%C5%A1ta-tiska-za-2019.pdf>.

Since political polarity is strongly present in everyday life in Croatia, it was also important to consider both right-wing and left-wing sources. For this reason, we decided to include one of the most visited right-wing online media, Dnevno.hr, thus completing a representative sample of eight online media.


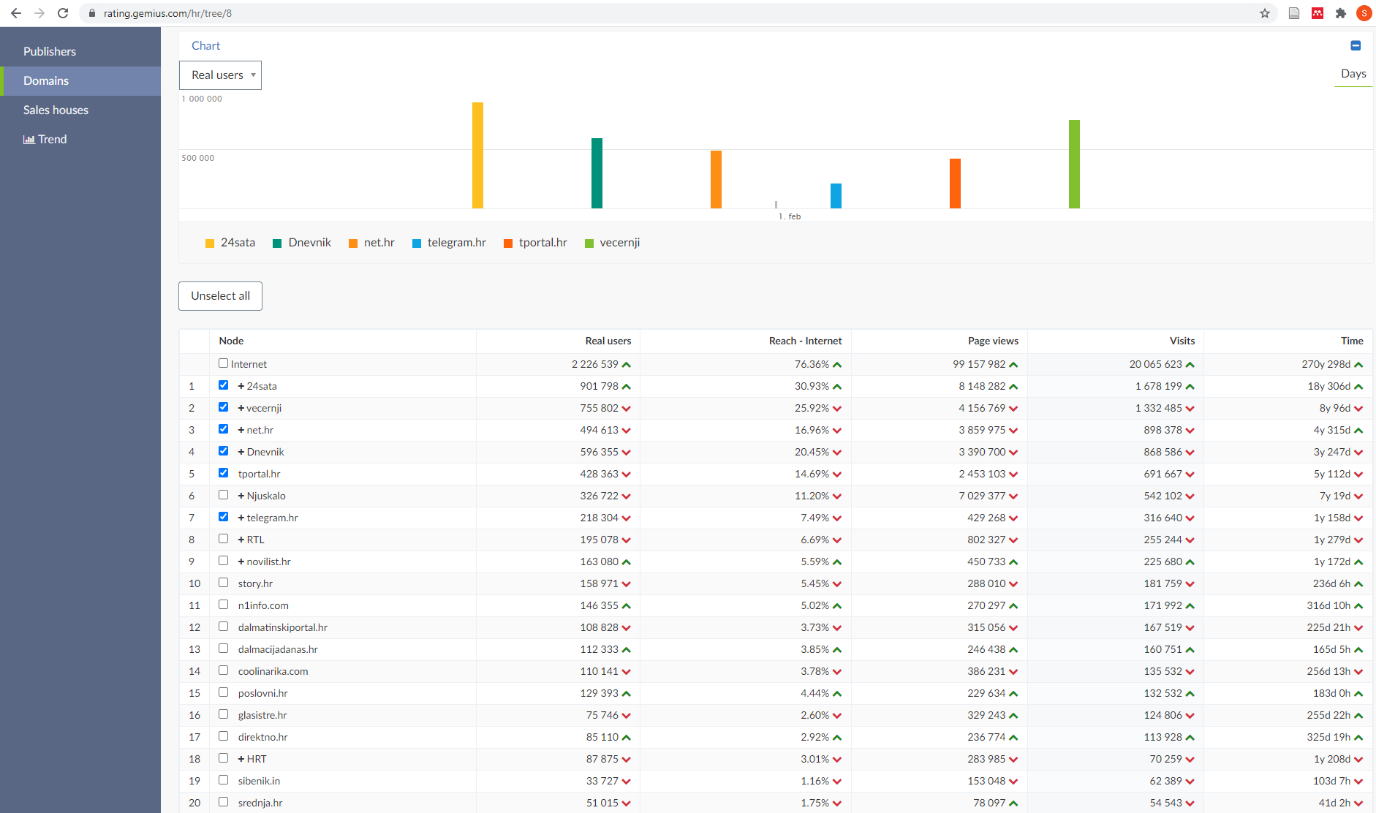


Figure A0-1: Gemius’ ranking list of online portals of various kind (retrieved from: <https://rating.gemius.com/hr>) – the blue marks indicate those among them that belong to the category of online news media, and were included in our study since they met the set criteria

**Section-A1: Definition and duration of epidemic waves**

An epidemic wave is a graph that tracks the number of people suffering from a disease over time. Epidemics usually begin with a sharp increase in the number of patients in a short time, that number then reaches a peak, after which it begins to decline until there are no new infections. Some epidemiological experts state that if there is no new case in a population for a certain number of days (e.g. 14 days), only then can the end of the epidemic (epidemic wave) be declared. The definition of a second wave is that the first wave must end and that a certain period must pass in between. In this study, there was no complete cessation for fourteen days without a single case of infection in Croatia. However, there was a lull of 25 days in which the number of infections was occasionally equal to zero, and occasionally one or two cases of infection occurred in the period from May 25, 2020 to June 16, 2020. The official date delimiting the two epidemic waves is not defined. Therefore, in this study we determined that the first wave lasted until May 15, 2020. After that, it starts a series of new infections with several infected cases that is greater than and equal to three. Due to the new sharp increase in new infections, we define June 15, 2020 as the beginning of the second epidemic wave.

**Section-A2: Tezaurus of COVID-19 Related Keywords**

List of the core COVID-19 related terms used in the classification of COVID-19 and non-COVID-19 articles (written in Croatian): *SARS-CoV-2, COVID-19, corona, korona, koronavirus, korona-virus, epidemija, pandemija, samoizolacija, novozaražen, koronakriza, propusnica, e-propusnica, cjepivo, cijepljenje, lockdown, WHO, stožer civilne zaštite, Capak, Beroš, Markotić.*

**Section-A3: Equations**

Cross-correlation function (CCF) was applied to quantify a potential association, and time lags between the two time series. ($X_{t}$ and $Y_{t}$ ), as shown in equation (1):

$CCF (l) = \frac{\sum_{t=1}^{n-l} (X_{t}-\bar{X})(Y_{t+l}\bar{Y})}{S_{x}S_{y}}$ (1)

where n is the number of observations, t represents row number, and lag is marked with *l*. Further, mean of X and Y are marked with $\underline{X}$ and $\underline{Y}$, while $S_{x}$ and $S_{y}$ are calculated by equations:

$S_{x}=\sqrt{\sum_{t=1}^{n} {{(X}_{t}-\bar{X})}^{2}}$ and $S_{y}=\sqrt{\sum_{t=1}^{n} {{(Y}_{t}-\bar{Y})}^{2}}$ (2).

The lag is the number of time periods that separate the two time series. In our experiments, the default number of lags ranges from ( $-\sqrt{n}+10$) to ($\sqrt{n}+10$).

The correlation is considered significant when the absolute value is greater than:

$\frac{2}{\sqrt{n-|l|}}$ (3).

This is a rule of thumb procedure based on large-sample normal approximation [1], [2].

The autocorrelation function for lag *l* can be calculated by the equation [3]:

$ACF(l)=\frac{\sum_{t=l+1}^{n-l} (X_{t-l}-\bar{X})(X_{t}-\bar{X})}{\sum_{t=1}^{n} {(X_{t}-\bar{X})}^{2}}$ (4)

where *l* denotes lag, $X_{t}$is value of X at row *t*,$\underline{X}$ is the mean of *X*, and *n* is the number of observations in the series.

PMI measured the level of dependency between two observations (X and Y) using equation:

$PMI(x, y) = log (\frac{p(x,y)}{p\left( x \right)p(y)}$) (5)

where p(.) is the probability function. The two observations which frequently co-occurred, or in other words were strongly associated with each other, had a high PMI value. The average dependency or MI between the two random variables X and Y can be calculated by the equation:

$MI\left( X,Y \right)=\sum_{i=1}^{|X|} \sum_{j=1}^{|Y|} \frac{|X_{i}\cap Y_{j}|}{n}log(\frac{n|X_{i}\cap Y_{j}|}{|X_{i}||Y_{j}|})$. (6)

The normalized mutual information is defined as

$NMI(X,Y)=\frac{MI(X,Y)}{mean(H\left( X \right),H(Y))}$ (7).

Jaccard similarity coefficient, also known as the Jaccard similarity index, is a [statistic](https://en.wikipedia.org/wiki/Statistic) measure used for expressing the [similarity](https://en.wikipedia.org/wiki/Similarity_measure) and [diversity](https://en.wikipedia.org/wiki/Diversity_index) of [sample](https://en.wikipedia.org/wiki/Sample_(statistics)) sets. For two sets *A* and *B*, the Jaccard similarity index is defined as the size of the [intersection](https://en.wikipedia.org/wiki/Intersection_(set_theory)) divided by the size of the [union](https://en.wikipedia.org/wiki/Union_(set_theory)) of sets *A* and *B*:

$J\left( A,B \right)=\frac{|A\cap B|}{|A\cup B|}$ (8).

Note that by design, $0\leq J(A,B)\leq1$. If A and B are both empty, then we define $J\left( A,B \right)=1$.  The higher values of Jaccard similarity index indicate the higher similarity between two sets of observed words (and vice versa) – i.e. similarity between the lists of the most frequent words from two different time intervals (e.g., two months or two epidemic waves).

**Section-A4: Results of Statistical Analysis of Correlations**

**Data distribution**


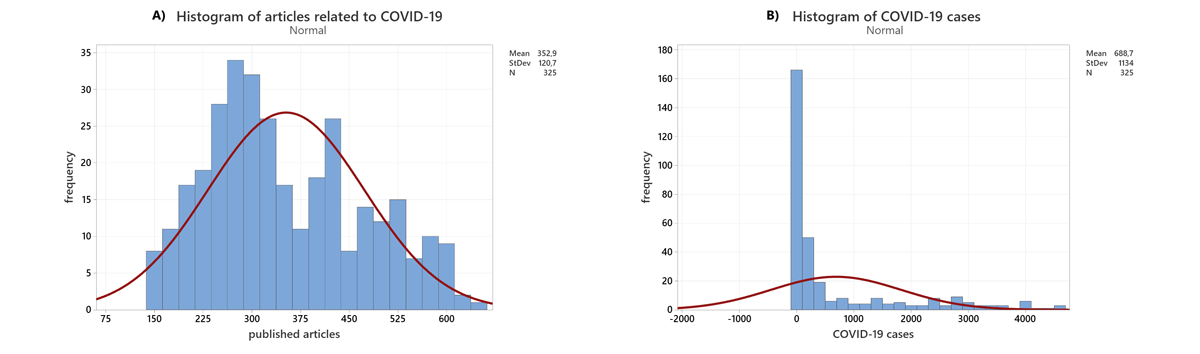
Figure A4-1. A histogram of frequencies for published COVID-19 articles per day (top-left), a histogram of frequencies for new COVID-19 cases in Croatia per day (top-right)

**Normality test for two time series data sampled per day**

In the preliminary analysis we notice that the observed data are not normally distributed, i.e. neither of them follow the Gaussian bell-shaped distribution. This is confirmed by the Kolmogorov-Smirnov test for normality.


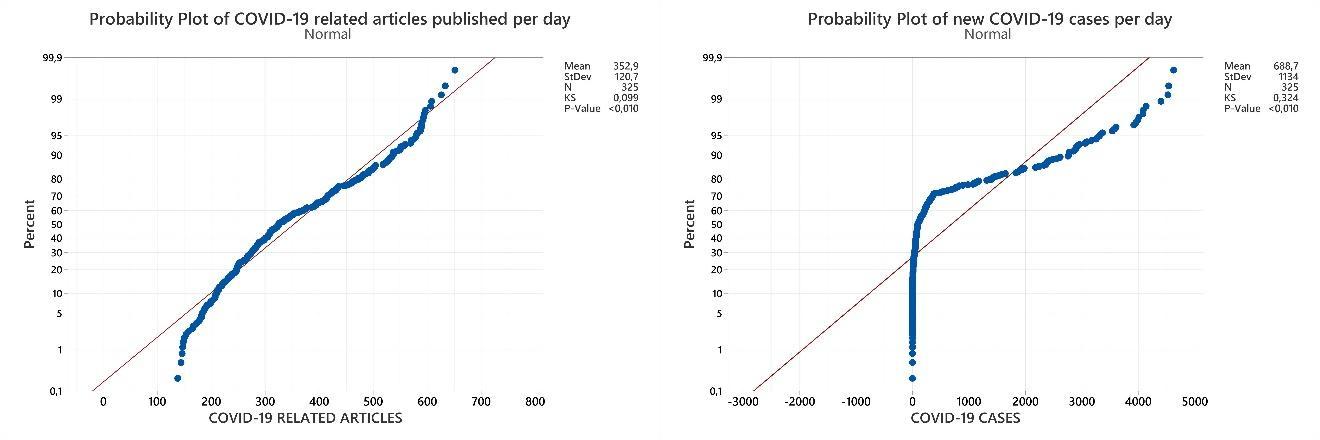


Figure A4-2. Probability plot of COVID-19 related articles published per day (left), and COVID-19 cases per day in the Republic of Croatia (right).

A Kolmogorov-Smirnov test indicates that the COVID-19 articles published per day do not follow a normal distribution, *D*(325) = .099, *P* < .001. A Kolmogorov-Smirnov test indicates that the COVID-19 cases per day do not follow a normal distribution, *D*(325) = .324, *P* < .001.

Table A4-1. Pairwise Spearman Correlations for time series data per day (with Confidence Intervals).

|  | $\rho$ | *P* (2-tailed) | 95% Confidence Intervals  (2-tailed)^a,b^ | |
| --- | --- | --- | --- | --- |
|  |  |  | Lower | Upper |
| Sample 1: COVID-19 articles Sample 2: COVID-19 cases N = 325 | .253 | <.001 | .145 | .355 |
| a. Estimation is based on Fisher's r-to-z transformation. | | | | |
| b. Estimation of standard error is based on the formula proposed by Fieller, Hartley, and Pearson.  Table A4-2. Pairwise Kendall's tau for time series data per day (with Confidence Intervals) | | | | |
|  | Kendall's $\tau$ | *P* (2-tailed) | 95% Confidence Intervals  (2-tailed)^a^ | |
|  |  |  | Lower | Upper |
| Sample 1: COVID-19 articles Sample 2: COVID-19 cases N = 325 | .173 | <.001 | .102 | .242 |
| a. Estimation is based on Fisher's r-to-z transformation. | | | | |

**Normality test for two time series data sampled per week (7 days)**

**
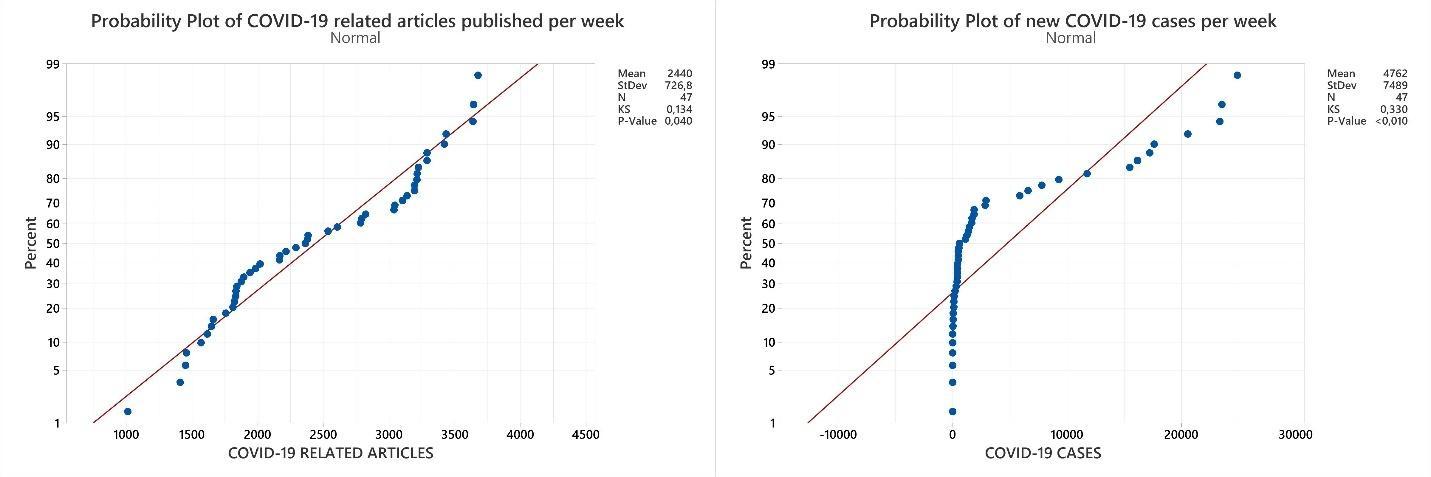
**

Figure A4-3. Probability plot of COVID-19 related articles published per week (left), and COVID-19 cases per week in the Republic of Croatia (right).

A Kolmogorov-Smirnov test indicates that the COVID-19 articles published per week do not follow a normal distribution, *D*(47) = .134, *P* < .050. A Kolmogorov-Smirnov test indicates that the COVID-19 cases per week do not follow a normal distribution, *D*(47) = .330, *P* < .010.

Table A4-4. Pairwise Spearman Correlations for time series data per week (with Confidence Intervals)

|  | $\rho$ | *P*  (2-tailed) | 95% Confidence Intervals (2-tailed)^a,b^ | |
| --- | --- | --- | --- | --- |
|  |  |  | Lower | Upper |
| Sample 1: COVID-19 articles Sample 2: COVID-19 cases N = 47 | .277 | .060 | -.020 | .529 |
| a. Estimation is based on Fisher's r-to-z transformation. | | | | |
| b. Estimation of standard error is based on the formula proposed by Fieller, Hartley, and Pearson. | | | | |

Table A4-5. Pairwise Kendall's tau for time series data per week (with Confidence Intervals).

|  | Kendall's $\tau$ | *P*  (2-tailed) | 95% Confidence Intervals (2-tailed)^a^ | |
| --- | --- | --- | --- | --- |
|  |  |  | Lower | Upper |
| Sample 1: COVID-19 articles Sample 2: COVID-19 cases N = 47 | .202 | .046 | .007 | .382 |
| a. Estimation is based on Fisher's r-to-z transformation. | | | | |

**Correlograms (autocorrelation function)**


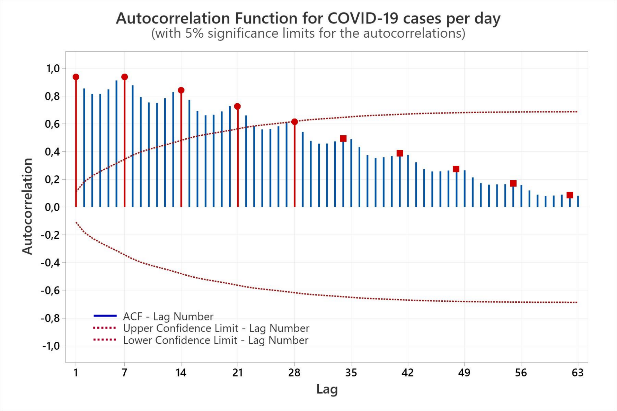

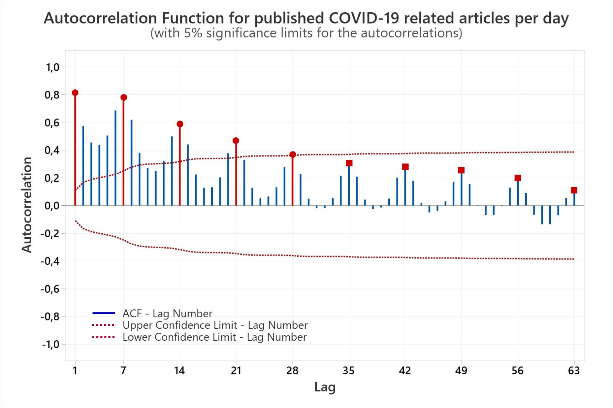


Figure A4-4. ACF plot for COVID-19 related articles per day (left), and COVID-19 cases per day in the Republic of Croatia (right).

Correlograms shows a plot of autocorrelation function (ACF) on a time series data of new COVID-19 cases per day (left plot), and plot of ACF on a time series data of published COVID-19 related articles per day by lag (right plot). This autocorrelation measures the linear relationship between lagged values of a time series.

**Mutual information results**

Table A4-6. The mutual information and normalized mutual information between COVID-19 cases per day and published COVID-19 related articles per day in online news media during the first and second waves, and 2020.

|  | **1^st^ wave** | **2^nd^ wave** | **2020 year** |
| --- | --- | --- | --- |
| **MI** | 3.2301 | 4.8498 | 4.6536 |
| **NMI** | 0.8145 | 0.9490 | 0.8917 |

In addition to CCF, to get as detailed insights as possible, we want precise results and their clear interpretation. Correlations give us an insight into the degree of linear association between two variables but say nothing about causality. However, with MI we complemented CCF and further quantified this association with a numeric value. We observed the mutual dependence of the two time series in the context of MI and NMI, as shown in Table A3-6. The highest MI and NMI occurred in the second pandemic wave in contrast to the first wave and the whole of 2020 in general. In the first wave, we may have expected a lot more newspaper news about the virus, which was new and unknown at the time, than we expected in the second wave, when something about the virus was already known and discovered. Nevertheless, MI and NMI reach higher values in the second wave. A possible explanation for such results is the fact that little was known about the virus in the first wave, and perhaps enough was written about it. In the second wave, almost every topic was covered by the admixture of information about the virus, whether it was about the economy, tourism, education, culture, sports or any other life topic. Besides, it is important to note that the highest MI occurred in the second pandemic wave when the total number of published news was slightly higher than in the first wave (with a difference of approximately 60,000 more articles). In addition to this, the number of COVID-19 infections also reached significantly higher daily frequencies in the second wave.

**Spearman correlations for 8 online portals**


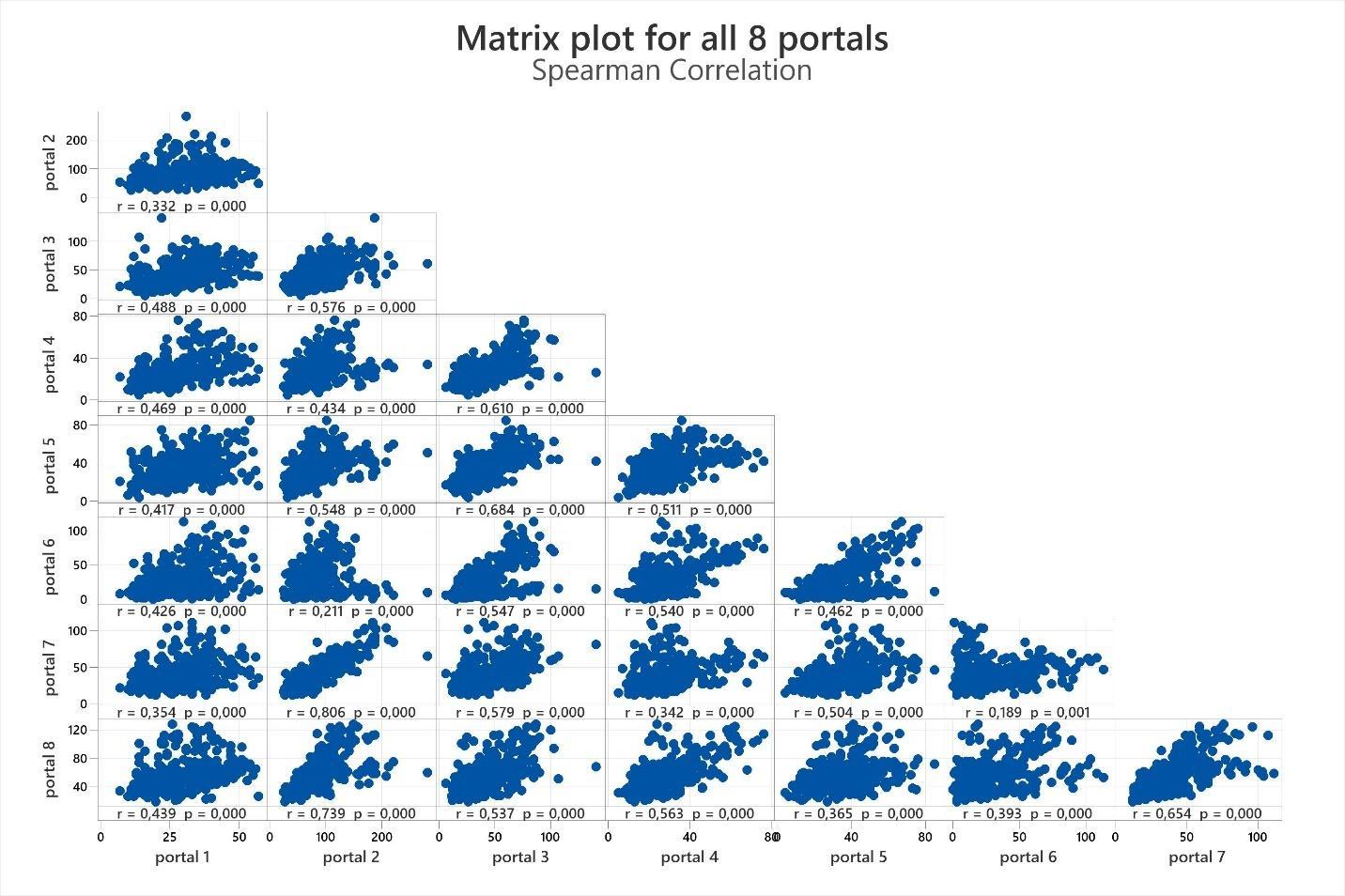


Figure A4-5. Matrix plot of eight news portals.

Table A4-7. Pairwise Spearman Correlations with 95% Confidence Intervals.

| Sample 1 – Sample 2  N=325 | $\rho$ | *P* (2-tailed) | 95% Confidence Intervals (2-tailed)^a,b^ | |
| --- | --- | --- | --- | --- |
|  |  |  | Lower | Upper |
| portal1 - portal2 | .332 | <.001 | .229 | .428 |
| portal1 - portal3 | .488 | <.001 | .397 | .569 |
| portal1 - portal4 | .469 | <.001 | .377 | .552 |
| portal1 - portal5 | .417 | <.001 | .320 | .506 |
| portal1 - portal6 | .426 | <.001 | .329 | .513 |
| portal1 - portal7 | .354 | <.001 | .252 | .448 |
| portal1 - portal8 | .439 | <.001 | .344 | .526 |
| portal2 - portal3 | .576 | <.001 | .496 | .646 |
| portal2 - portal4 | .434 | <.001 | .338 | .521 |
| portal2 - portal5 | .548 | <.001 | .464 | .622 |
| portal2 - portal6 | .211 | <.001 | .101 | .315 |
| portal2 - portal7 | .806 | <.001 | .763 | .842 |
| portal2 - portal8 | .739 | <.001 | .684 | .786 |
| portal3 - portal4 | .610 | <.001 | .534 | .676 |
| portal3 - portal5 | .684 | <.001 | .619 | .739 |
| portal3 - portal6 | .547 | <.001 | .463 | .621 |
| portal3 - portal7 | .579 | <.001 | .499 | .649 |
| portal3 - portal8 | .537 | <.001 | .452 | .612 |
| portal4 - portal5 | .511 | <.001 | .423 | .589 |
| portal4 - portal6 | .540 | <.001 | .455 | .615 |
| portal4 - portal7 | .342 | <.001 | .239 | .437 |
| portal4 - portal8 | .563 | <.001 | .481 | .635 |
| portal5 - portal6 | .462 | <.001 | .369 | .545 |
| portal5 - portal7 | .504 | <.001 | .415 | .583 |
| portal5 - portal8 | .365 | <.001 | .264 | .459 |
| portal6 - portal7 | .189 | <.001 | .078 | .294 |
| portal6 - portal8 | .393 | <.001 | .294 | .484 |
| portal7 - portal8 | .654 | <.001 | .585 | .714 |
| a. Estimation is based on Fisher's r-to-z transformation. | | | | |
| b. Estimation of standard error is based on the formula proposed by Fieller, Hartley, and Pearson.  Table A4-8. Pairwise Kendall's tau with 95% Confidence Intervals. | | | | |
| Sample 1 – Sample 2  N=325 | Kendall's $\tau$ | *P* (2-tailed) | 95% Confidence Intervals (2-tailed)^a^ | |
|  |  |  | Lower | Upper |
| portal1 - portal2 | .224 | <.001 | .154 | .291 |
| portal1 - portal3 | .339 | <.001 | .273 | .401 |
| portal1 - portal4 | .329 | <.001 | .263 | .392 |
| portal1 - portal5 | .300 | <.001 | .233 | .364 |
| portal1 - portal6 | .298 | <.001 | .231 | .363 |
| portal1 - portal7 | .237 | <.001 | .168 | .304 |
| portal1 - portal8 | .304 | <.001 | .237 | .368 |
| portal2 - portal3 | .408 | <.001 | .346 | .466 |
| portal2 - portal4 | .307 | <.001 | .241 | .371 |
| portal2 - portal5 | .382 | <.001 | .319 | .442 |
| portal2 - portal6 | .156 | <.001 | .084 | .225 |
| portal2 - portal7 | .623 | <.001 | .577 | .665 |
| portal2 - portal8 | .555 | <.001 | .503 | .603 |
| portal3 - portal4 | .444 | <.001 | .384 | .500 |
| portal3 - portal5 | .488 | <.001 | .431 | .541 |
| portal3 - portal6 | .405 | <.001 | .343 | .464 |
| portal3 - portal7 | .415 | <.001 | .353 | .473 |
| portal3 - portal8 | .378 | <.001 | .315 | .438 |
| portal4 - portal5 | .360 | <.001 | .295 | .421 |
| portal4 - portal6 | .387 | <.001 | .324 | .447 |
| portal4 - portal7 | .238 | <.001 | .169 | .305 |
| portal4 - portal8 | .410 | <.001 | .348 | .468 |
| portal5 - portal6 | .341 | <.001 | .276 | .403 |
| portal5 - portal7 | .346 | <.001 | .281 | .408 |
| portal5 - portal8 | .249 | <.001 | .181 | .316 |
| portal6 - portal7 | .137 | <.001 | .066 | .208 |
| portal6 - portal8 | .275 | <.001 | .207 | .341 |
| portal7 - portal8 | .470 | <.001 | .412 | .525 |
| a. Estimation is based on Fisher's r-to-z transformation. | | | | |

**Section-A5: Results of** **coronavirus related concepts analysis**

Table A5-1. Analysis of the most frequent terminology by waves represented by TOP 50 most frequent unigrams on the lemmatized corpus (translated from Croatian language). Coloring helps in identifying seven thematic groups of related sets of words: neutral, medical, time, geo-spatial, quantitative, governing or economy.

|  | **WAVE 1** | | | **WAVE 2** | | | |
| --- | --- | --- | --- | --- | --- | --- | --- |
| 1 | man/person | 112477 | | man/person | | 211435 | |
| 2 | coronavirus | 87358 | | Croatia | | 120513 | |
| 3 | Croatia | 85450 | | year | | 114570 | |
| 4 | year | 69040 | | new | | 106472 | |
| 5 | measure | 61019 | | large | | 88352 | |
| 6 | day | 60861 | | measure | | 82728 | |
| 7 | large | 60484 | | day | | 81496 | |
| 8 | new | 54398 | | coronavirus | | 78014 | |
| 9 | epidemic/pandemic | 49318 | | number | | 77262 | |
| 10 | infection | 46616 | | infection | | 66928 | |
| 11 | to work | 41485 | | case | | 66832 | |
| 12 | number | 41465 | | epidemic/pandemic | | 56751 | |
| 13 | case | 40736 | | COVID-19 | | 55237 | |
| 14 | time | 36266 | | percent | | 54358 | |
| 15 | country | 34884 | | county | | 52978 | |
| 16 | virus | 34500 | | clock/hour | | 51702 | |
| 17 | percent | 33308 | | to work | | 50526 | |
| 18 | government | 32383 | | time | | 44532 | |
| 19 | situation | 32370 | | government | | 44234 | |
| 20 | good | 27877 | | country | | 44146 | |
| 21 | headquarters | 25571 | | good | | 41021 | |
| 22 | state (država) | 25012 | | territory | | 40843 | |
| 23 | clock/hour | 24559 | | headquarters | | 37643 | |
| 24 | city | 23928 | | vaccine | | 37119 | |
| 25 | minister | 23454 | | situation | | 37093 | |
| 26 | week | 23149 | | president | | 35391 | |
| 27 | citizen | 22789 | | city | | 35154 | |
| 28 | Zagreb | 22773 | | hospital | | 34818 | |
| 29 | working | 22747 | | virus | | 34141 | |
| 30 | public | 22285 | | positive | | 33795 | |
| 31 | protection | 22108 | | state (država) | | 32800 | |
| 32 | hospital | 21925 | | milijun | | 32141 | |
| 33 | health care (zdravstven) | 21891 | | 24 | | 31357 | |
| 34 | decision | 21643 | | week | | 31063 | |
| 35 | world | 21626 | | self-isolationa | | 29933 | |
| 36 | president | 21037 | | Zagreb | | 29899 | |
| 37 | COVID-19 | 20868 | | last | | 29897 | |
| 38 | month | 20755 | | public | | 28679 | |
| 39 | job | 20635 | | month | | 28141 | |
| 40 | disease | 20453 | | European | | 28119 | |
| 41 | million | 20448 | | health care (zdravstven) | | 27903 | |
| 42 | question | 20145 | | minister | | 27515 | |
| 43 | county | 19835 | | citizen | | 27382 | |
| 44 | European | 19561 | | question | | 27108 | |
| 45 | house | 19180 | | disease | | 26330 | |
| 46 | kuna | 18783 | | protection | | 25985 | |
| 47 | crisis | 18752 | | working | | 25758 | |
| 48 | far | 18679 | | patient | | 25628 | |
| 49 | home | 18312 | | decision | | 25616 | |
| 50 | life | 18115 | | kuna | | 25196 | |
|  |  |  | |  | |  | |
|  | medical |  | quantitative | |  | |  |
|  | geo-spatial |  | governing | |  | |  |
|  | time |  | economy | |  | |  |
|  | neutral |  |  | |  | |  |

The complete list of drugs and vaccines included in terminology analysis of this study, related to group of words naming drugs and vaccines, are listed in Table A5-2. In the experiment, list of lemmas were used instead of words. It is important to note that list of lemmas for a group of terms that name vaccines contain not only the names of vaccines but also the names of their producers.

Table A5-2. List of drugs and vaccines included in terminology analysis.

| **DRUGS** | **VACCINES** |
| --- | --- |
| *aspirin*  *sumamed*  *azitromicin*  *paracetamol*  *hidroklorokin*  *lopinavir*  *ritonavir*  *deksametazon*  *remdesivir* | *novavax*  *curevac*  *sputnjik V*  *sinovac*  *sanofi*  *pfizer*  *astrazeneca*  *biontech*  *janssen*  *moderna*  *comirnaty*  *gamaleya* |

**Section-A6: NER Results**

The list of recognised entities divided into 4 categories, persons (PER), location (LOC), organization (ORG) and others (MISC), are distributed into separate files categorized by months, and are available from <https://github.com/sbeliga/InfoCoV/tree/main/JMIR/NER>.

**References**

[1] “Minitab tutorial.” [Online]. Available: https://support.minitab.com/en-us/minitab/18/help-and-how-to/modeling-statistics/time-series/how-to/cross-correlation/interpret-the-results/all-statistics-and-graphs/

[2] “Minitab tutorial (vol2).” [Online]. Available: https://support.minitab.com/en-us/minitab/19/help-and-how-to/statistical-modeling/time-series/how-to/cross-correlation/methods-and-formulas/methods-and-formulas/

[3] “Minitab tutorial: Methods and formulas for Autocorrelation.” [Online]. Available: https://support.minitab.com/en-us/minitab/18/help-and-how-to/modeling-statistics/time-series/how-to/autocorrelation/methods-and-formulas/methods-and-formulas/
